# Identification of novel single nucleotide variants in the drug resistance mechanism of *Mycobacterium tuberculosis* isolates by whole-genome analysis

**DOI:** 10.1186/s12864-024-10390-3

**Published:** 2024-05-14

**Authors:** Weiye Qian, Nan Ma, Xi Zeng, Mai Shi, Mingqiang Wang, Zhiyuan Yang, Stephen Kwok-Wing Tsui

**Affiliations:** 1 School of Artificial Intelligence, Hangzhou Dianzi University, Hangzhou, 310018 China; 2 Agricultural Bioinformatics Key Laboratory of Hubei Province and 3D Genomics Research Centre, College of Informatics, Huazhong Agricultural University, Wuhan, 430070 China; 3 School of Biomedical Sciences, The Chinese University of Hong Kong, Hong Kong SAR, China; 4 Stanford Cardiovascular Institute, Stanford University School of Medicine, Stanford, CA 94305 USA; 5 Hong Kong Bioinformatics Centre, The Chinese University of Hong Kong, Hong Kong SAR, China

**Keywords:** *Mycobacterium tuberculosis*, Single nucleotide variant, Whole-genome sequencing

## Abstract

**Background:**

Tuberculosis (TB) represents a major global health challenge. Drug resistance in *Mycobacterium tuberculosis* (MTB) poses a substantial obstacle to effective TB treatment. Identifying genomic mutations in MTB isolates holds promise for unraveling the underlying mechanisms of drug resistance in this bacterium.

**Methods:**

In this study, we investigated the roles of single nucleotide variants (SNVs) in MTB isolates resistant to four antibiotics (moxifloxacin, ofloxacin, amikacin, and capreomycin) through whole-genome analysis. We identified the drug-resistance-associated SNVs by comparing the genomes of MTB isolates with reference genomes using the MuMmer4 tool.

**Results:**

We observed a strikingly high proportion (94.2%) of MTB isolates resistant to ofloxacin, underscoring the current prevalence of drug resistance in MTB. An average of 3529 SNVs were detected in a single ofloxacin-resistant isolate, indicating a mutation rate of approximately 0.08% under the selective pressure of ofloxacin exposure. We identified a set of 60 SNVs associated with extensively drug-resistant tuberculosis (XDR-TB), among which 42 SNVs were non-synonymous mutations located in the coding regions of nine key genes (ctpI, desA3, mce1R, moeB1, ndhA, PE_PGRS4, PPE18, rpsA, secF). Protein structure modeling revealed that SNVs of three genes (PE_PGRS4, desA3, secF) are close to the critical catalytic active sites in the three-dimensional structure of the coding proteins.

**Conclusion:**

This comprehensive study elucidates novel resistance mechanisms in MTB against antibiotics, paving the way for future design and development of anti-tuberculosis drugs.

**Supplementary Information:**

The online version contains supplementary material available at 10.1186/s12864-024-10390-3.

## Introduction

Tuberculosis (TB), primarily caused by *Mycobacterium tuberculosis* (MTB), is one of the major epidemics worldwide, with a high mortality rate surpassing that of any other infectious disease [1]. In 2021, the World Health Organization estimated that around 10 million people were affected by tuberculosis, leading to 1.6 million deaths [2]. TB is a treatable and curable disease, often managed through a combination of antibiotics. However, a critical challenge in TB treatment lies in the widespread drug-resistance mechanisms manifested by MTB [3].

Drug-resistant tuberculosis can be classified into various categories, including multidrug-resistant tuberculosis (MDR-TB) and extensively drug-resistant tuberculosis (XDR-TB) [4]. MDR-TB exhibits resistance to isoniazid and rifampicin, while XDR-TB exhibits resistance to all first-line drugs and at least one second-line drug [5]. Gupta et al. reported that ofloxacin resistance is significantly high among multidrug-resistant MTB strains across India, most of which were especially associated with the Beijing genotype and carried gyrA mutations [6]. Fluoroquinolone is the classical representative of first-line drugs in TB treatment. MTB acquires resistance to fluoroquinolones mainly through mutations in the quinolone resistance-determining region of the pknB gene [7]. Moxifloxacin is a common second-line drug in the treatment of pneumonia and tuberculosis infections [8]. Clinical trials have shown that moxifloxacin can improve standard treatment regimens with better bactericidal activity [9]. Understanding the mechanisms of drug resistance in MTB is crucial for developing new treatment strategies and improving the management of drug-resistant TB cases.

MTB is characterized by several genomic features that contribute to its adaptability, virulence, and ability to survive within host organisms. In 2013, the reference genome assembly ASM19595v2 of MTB H37Rv was published. Within the genomic landscape of MTB, high-frequency genomic mutations play pivotal roles in drug-resistance mechanisms [10]. In our previous studies, we have identified the function of hypothetical proteins in the MTB genome and explored the drug-resistance mechanism of insertions and deletions in 1110 MTB isolates [11, 12]. In bacteria, gene mutations can contribute to genetic diversity and lead to the emergence of strains resistant to specific drugs. For instance, it was reported that mutations in gene rpoB, which codes for the RNA polymerase beta subunit, were associated with rifampin resistance in MTB [13]. Mutations in gene katG were linked to isoniazid resistance [14]. Thus, investigation of genomic mutations in drug-resistant isolates could help uncover novel drug-resistance mechanisms in MTB.

Single nucleotide variant (SNV) is a mutation at a single position of the genome sequence, often used as a marker to study the association between drug-resistance features [15]. SNVs can be divided into synonymous mutations and non-synonymous mutations, according to their functional consequences. Non-synonymous mutations are genetic variations that result in a change to the amino acid sequence of the encoded protein, potentially altering the structure and function of the protein.

In this study, we analyzed the whole genome of 716 clinical MTB isolates to identify the non-synonymous SNVs in XDR strains. We determined SNVs associated with drug resistance by comparing the whole genomes of XDR isolates against the MTB reference genome. We compared the frequencies of resistant and susceptible strains for different antibiotics using Fisher’s exact test. The non-synonymous mutations in some key genes were reported and discussed. Our findings suggested a novel view of drug-resistance mechanisms in MTB.

## Results

### WGS data analysis on antibiotic resistance

**Fig. 1:**
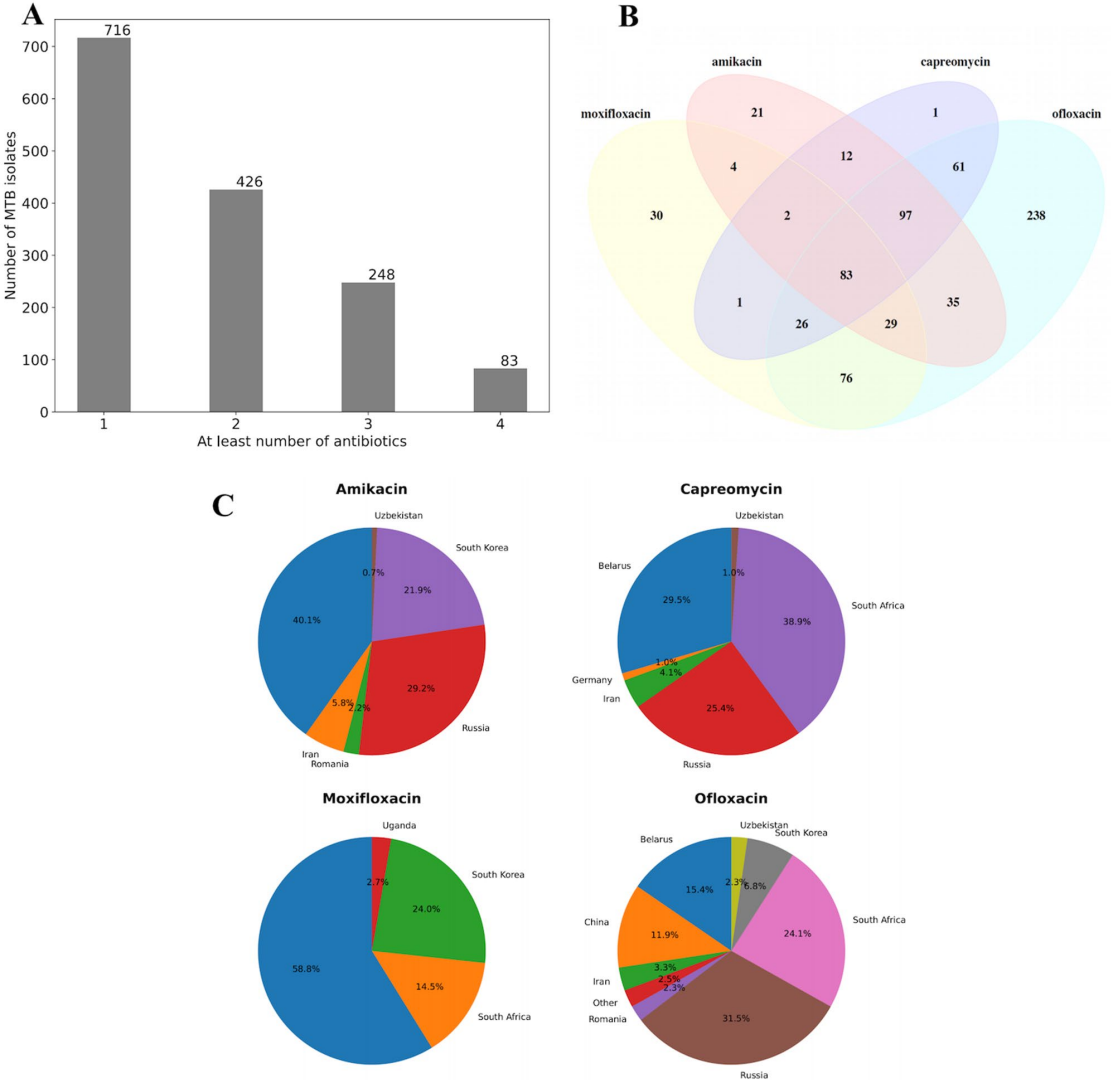
Whole-genome sequencing samples obtained in this study. (**A**) Number of MTB isolates tested by at least 1, 2, 3, or 4 antibiotics; (**B**) Venn diagram of samples tested by four antibiotics; (**C**) prevalence of drug resistance

Our focus centered on exploring the resistance mechanisms of MTB isolates resistant to two fluoroquinolones, namely moxifloxacin and ofloxacin, as well as two second-line drugs, amikacin and capreomycin. The whole-genome sequences of 716 MTB isolates which were tested with at least one drug mentioned above were collected from the BV-BRC database (Fig. 1). Resistance to different drugs varies significantly between geographical locations. For example, for amikacin, Belarus, Russia, and South Korea show relatively high resistance, accounting for 40.01%, 29.2%, and 21.9% respectively, while Iran, Romania, and Uzbekistan have lower resistance. Other drugs such as capreomycin, moxifloxacin, and ofloxacin showed similar trends (Fig. 1C). In this study, a group of 83 MTB isolates were tested by all four antibiotics. The testing results revealed that a set of 251 MTB isolates exhibited resistance to moxifloxacin, while a larger set of 645 isolates demonstrated resistance to ofloxacin (Table 1). Additionally, 283 MTB isolates exhibited resistance to amikacin, and the same number of MTB isolates displayed resistance to capreomycin. The isolates resistant to ofloxacin accounted for the highest proportion, with 94.2% in testing samples, indicating that MTB is more prone to mutations induced by exposure to ofloxacin.

**Table 1:** Summary of testing results of drug resistance in obtained MTB isolates

AMR result	moxifloxacin		ofloxacin		amikacin		capreomycin	
	number	proportion	number	proportion	number	proportion	number	proportion
Susceptible	90	26.4%	40	5.8%	234	45.3%	241	46.0%
Resistant	251	73.6%	645	94.2%	283	54.7%	283	54.0%
Total	341	/	685	/	517	/	524	/

### SNV calling by MuMer4

**Fig. 2:**
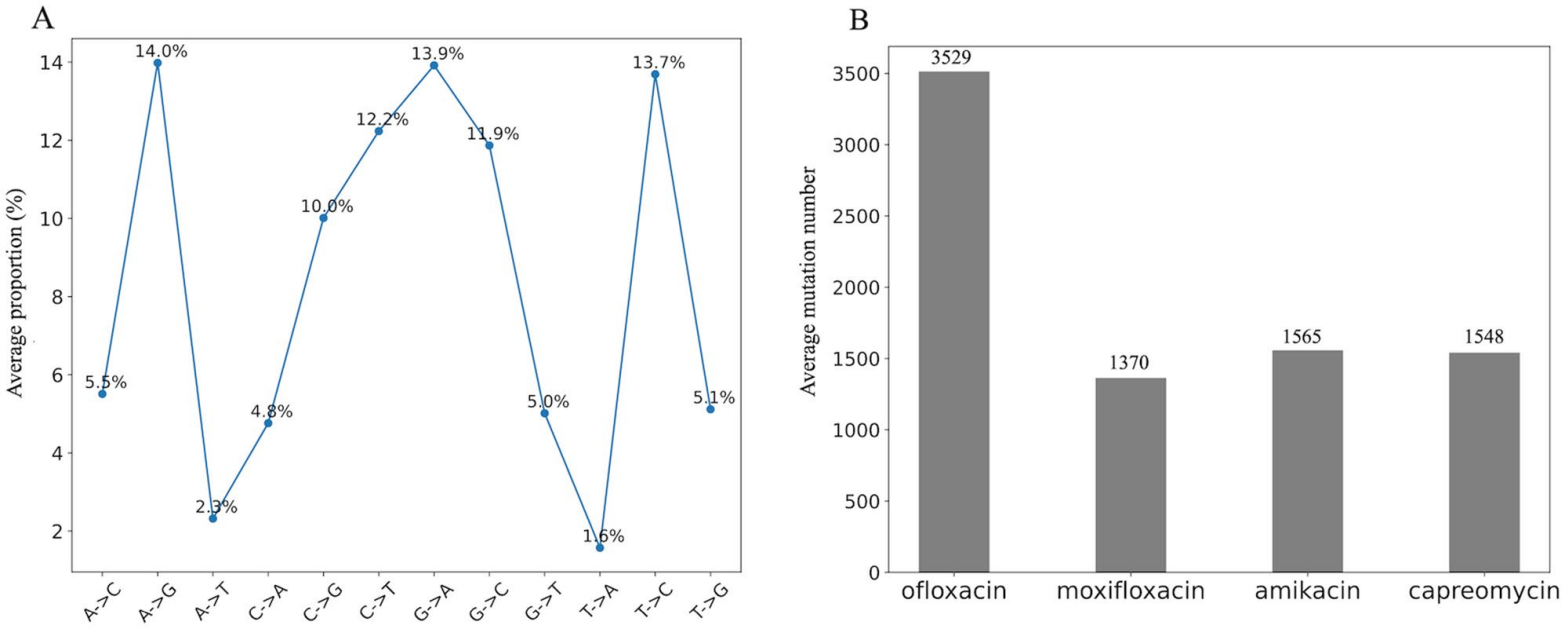
Mutation summary in antibiotic-resistant isolates. (**A**) Average proportion of different mutations in total amount; (**B**) Average mutation number in MTB isolates resistant to four different antibiotics

The WGS data of MTB isolates resistant to antibiotics were compared against the H37Rv reference genome using MUMmer4 to identify possible SNVs. The frequency and proportion of SNVs in MTB isolates resistant to the four antibiotics are also calculated (Table 2). Frequency represents the count of a certain mutation that occurs, while proportion represents the proportion of this mutation in the total count of mutations. As a result, we found that the mutation patterns were similar in the MTB isolates resistant to the four antibiotics. Notably, the proportions of T-> A mutations were consistently low across all antibiotics, accounting for only 1.6%, 1.53%, 1.55%, and 1.61%, respectively (Fig. 2A). In contrast, A-> G mutations were the most predominant, which accounted for around 14% of isolates resistant to three antibiotics (ofloxacin, moxifloxacin, and amikacin). Proportions of all the other mutations fell within the range of 2%-14%.

The average mutation number of the four antibiotics is presented in Fig. 2B. The greatest number of mutations were found in the ofloxacin-resistant MTB (an average of 3529 mutations in each MTB isolate). The fewest mutation number was found in the moxifloxacin-resistant MTB (an average of 1370 mutations in each MTB isolate). Considering the size of MTB genome (4,411,532 base pairs [16]), we estimated the mutation rate of one base as 0.080% in ofloxacin-resistant isolates and 0.031% in moxifloxacin-resistant MTB.

**Table 2:** Frequency and proportion of identified SNV mutations in MTB isolates

Mutation	ofloxacin		moxifloxacin		amikacin		capreomycin	
	Frequency	Proportion	Frequency	Proportion	Frequency	Proportion	Frequency	Proportion
A-> C	137,156	5.43%	54,640	5.57%	62,599	5.59%	60,421	5.45%
A-> G	352,580	13.96%	137,603	14.03%	156,779	13.99%	154,535	13.94%
A-> T	59,029	2.34%	22,712	2.31%	25,725	2.30%	25,842	2.33%
C-> A	120,528	4.77%	46,927	4.78%	53,364	4.76%	52,592	4.74%
C-> G	253,194	10.02%	98,347	10.02%	111,830	9.98%	111,208	10.03%
C-> T	309,840	12.26%	118,988	12.13%	137,574	12.28%	135,968	12.27%
G-> A	352,828	13.97%	135,914	13.85%	156,179	13.94%	154,143	13.91%
G-> C	299,840	11.87%	116,640	11.89%	132,730	11.85%	131,494	11.86%
G-> T	126,823	5.02%	49,249	5.02%	55,983	5.00%	55,592	5.02%
T-> A	40,413	1.60%	15,031	1.53%	17,326	1.55%	17,810	1.61%
T-> C	346,096	13.70%	134,863	13.75%	152,726	13.63%	151,806	13.70%
T-> G	128,097	5.07%	50,199	5.12%	57,554	5.14%	56,978	5.14%

### SNV screening by Fisher’s exact test

**Fig. 3:**
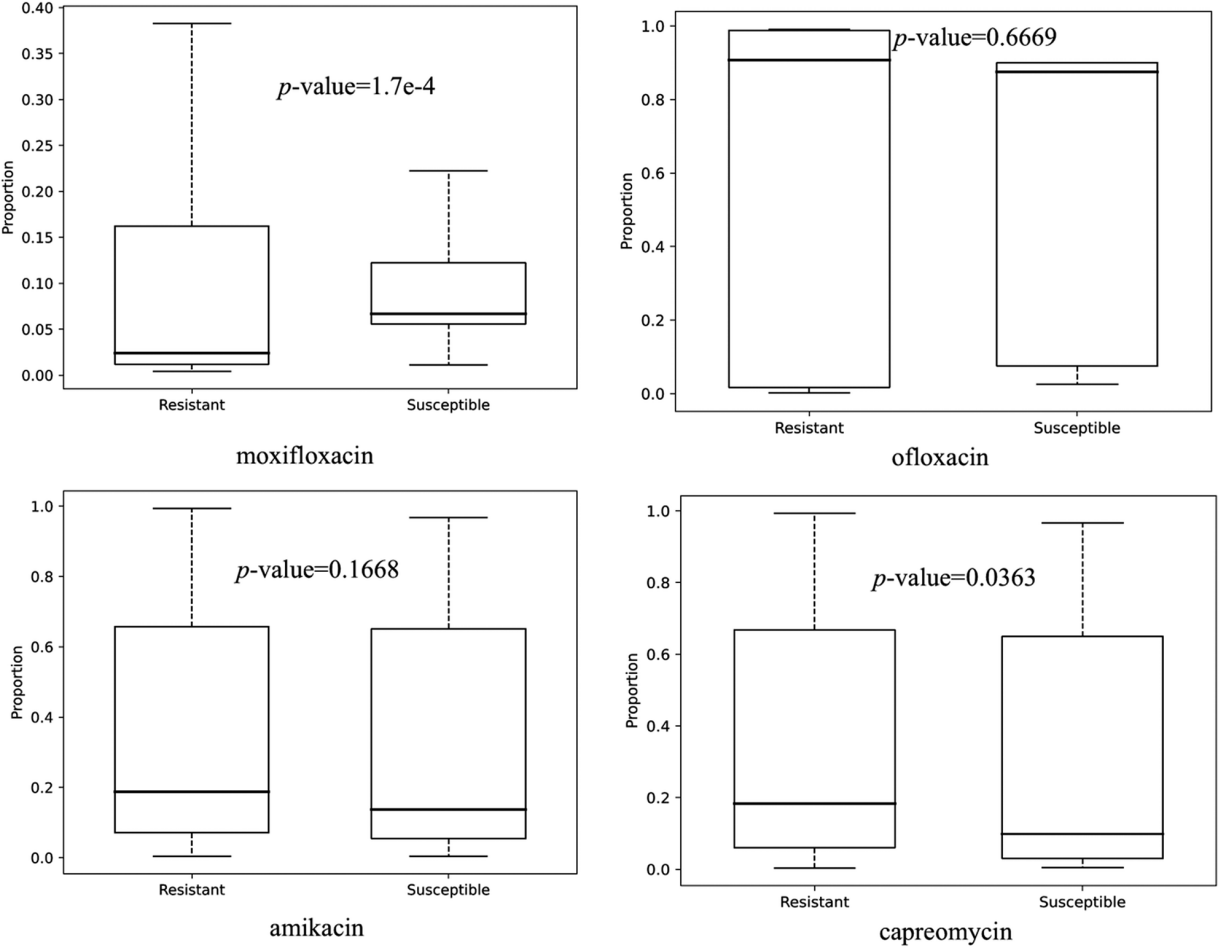
Box plot of frequency rate of SNVs in susceptible and resistant MTB isolates of four drugs

After identifying mutations in MTB, we employed Fisher’s exact test to determine SNVs that distributed differently between resistant and susceptible strains. The detailed number of significant SNVs and the type of mutation for each antibiotic are presented in Table 3. The number of non-synonymous (NS) mutations is larger than that of the synonymous mutations in all MTB isolates. The proportion of NS mutations is around 70% in three antibiotics (moxifloxacin, ofloxacin, amikacin) and 66.8% in capreomycin. A total of 3,536 and 3,541 NS mutations were found in MTB isolates resistant to moxifloxacin and ofloxacin, respectively. In addition, the frequency of screened SNVs showed significant differences in distribution between moxifloxacin-susceptible and moxifloxacin-resistant strains (*P* ≤ 0.001), but such differential distribution was not observed in the other three strains (Fig. 3). In moxifloxacin-resistant strains, the mutation frequency ranged from 0% to 20%, while in moxifloxacin-susceptible strains, it spanned from 5% to 15%. The median mutation frequency in susceptible strains surpassed that in resistant strains. These results indicated that moxifloxacin pressure induced more NS mutations and affected the evolution mechanism in MTB isolates.

**Table 3:** Distribution of mutation types of identified SNVs in MTB isolates resistant to each antibiotic

Mutation number	moxifloxacin		ofloxacin		amikacin		capreomycin	
	number	proportion	number	proportion	number	proportion	number	proportion
Synonymous mutation	1460	29.2%	1485	29.5%	517	29.9%	951	33.2%
NS mutation	3536	70.8%	3541	70.5%	1213	70.1%	1913	66.8%
Total	4996	100.0%	5026	100.0%	1730	100.0%	2864	100.0%

### Common SNVs related to four antibiotics

**Fig. 4:**
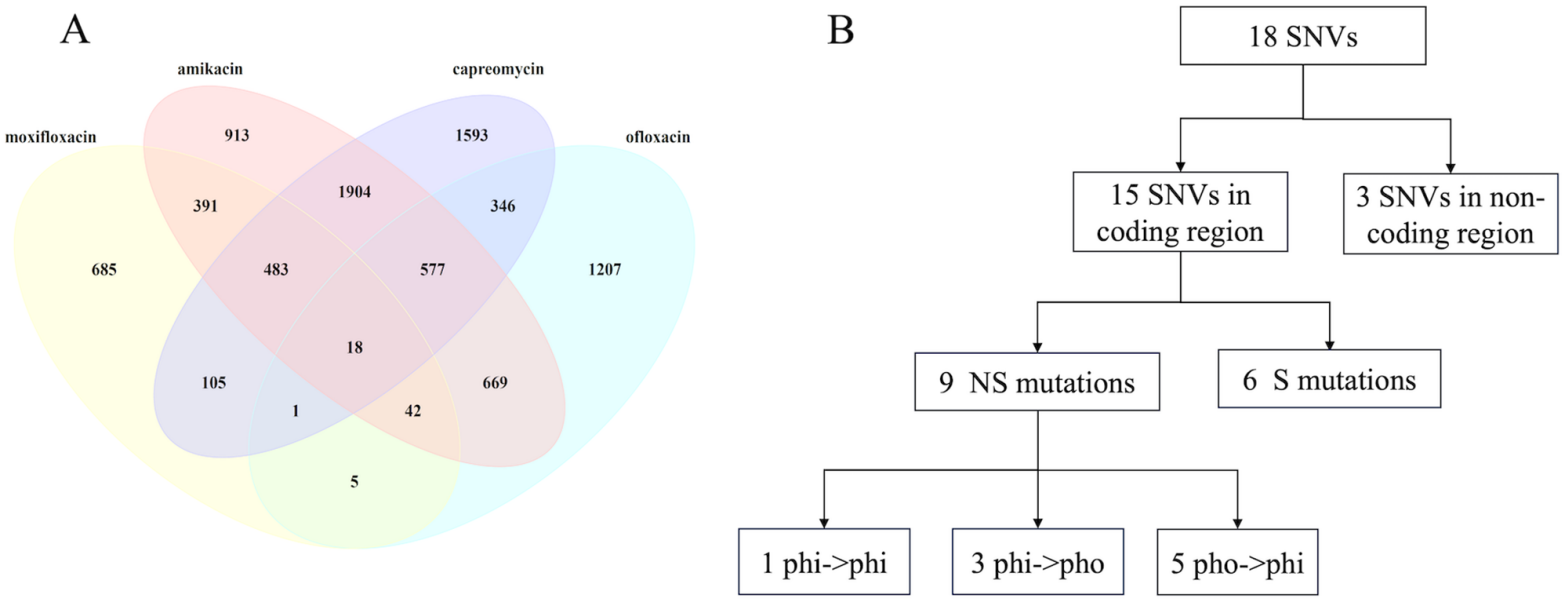
Common SNVs in MTB isolates are resistant to four antibiotics. (**A**) Venn diagram of common SNVs in MTB isolates resistant to different drugs; (**B**) Hydrophilic and hydrophobic analysis of translated amino acid of common SNVs.

By overlap analysis of Venn diagram, a small set of 18 significant SNVs was found in MTB strains resistant to all four antibiotics, accounting for 0.2% of total identified SNVs (Fig. 4A). The details of these 18 significant SNVs are detailed in Table 4. Among these, three SNVs (A300922G, T3379432C, T3381356C) are located in non-coding regions. Another SNV T103849C located in the coding region of Rv0094c, which encoded a hypothetical protein (accession number NP_214608.1). Information of codon mutations and amino acid mutations was also obtained. The rest 14 significant SNVs were specifically enriched in the coding region of gene PE_PGRS4. Among them, nine SNVs were NS mutations that cause amino acid changes in the encoded protein and five SNVs were synonymous mutations. Of the nine NS mutations, five SNV mutations resulted in an amino acid change from hydrophobic (pho) AA to hydrophilic (phi) AA, while three SNVs led to a reverse mutation from phi AA to pho AA (Fig. 4B). These results suggest that most SNVs could change the physiochemical characteristics of coding amino acids.

**Table 4:** Details of 18 common significant SNVs in MTB isolates resistant to four antibiotics. AA: amino acid; S: synonymous; NS: non-synonymous; pho: hydrophobic; phi: hydrophilic; Ref: nucleotide of the reference genome; Alt: nucleotide of the alternative genome

No.	RefSeq position	Ref	Alt	Coding region	Related gene	Mutation type	Codon mutation	AA mutation	Hydrophilic change
1	103,840	T	C	Yes	Rv0094c	NS	TGG-> CGG	Trp-> Arg	pho-> phi
2	338,511	C	A	Yes	PE_PGRS4	NS	GCC-> GAC	Ala-> Asp	pho-> phi
3	338,512	G	A	Yes	PE_PGRS4	NS	GCC-> ACC	Ala-> Thr	pho-> phi
4	338,530	G	T	Yes	PE_PGRS4	NS	GCC-> TCC	Ala-> Ser	pho-> phi
5	338,552	C	G	Yes	PE_PGRS4	NS	CCC-> CGC	Pro-> Arg	pho-> phi
6	338,553	G	T	Yes	PE_PGRS4	NS	AGG-> ATG	Arg-> Met	phi-> pho
7	338,554	A	G	Yes	PE_PGRS4	NS	AGG-> GGG	Arg-> Gly	phi-> pho
8	338,538	C	T	Yes	PE_PGRS4	NS	ACC-> ATC	Thr-> Ile	phi-> pho
9	338,544	C	G	Yes	PE_PGRS4	NS	ACG-> AGG	Thr-> Arg	phi-> phi
10	338,492	G	C	Yes	PE_PGRS4	S	GGG-> GGC	Gly-> Gly	
11	338,513	G	C	Yes	PE_PGRS4	S	GGG-> GGC	Gly-> Gly	
12	338,522	C	A	Yes	PE_PGRS4	S	GGC-> GGA	Gly-> Gly	
13	338,531	C	G	Yes	PE_PGRS4	S	GGC-> GGG	Gly-> Gly	
14	338,543	G	T	Yes	PE_PGRS4	S	ACG-> ACT	Thr-> Thr	
15	338,561	C	T	Yes	PE_PGRS4	S	GCC-> GCT	Ala-> Ala	
16	39,022	A	G	No					
17	3,379,432	T	C	No					
18	3,381,356	T	C	No					

### Common genes in XDR-TB

**Fig. 5:**
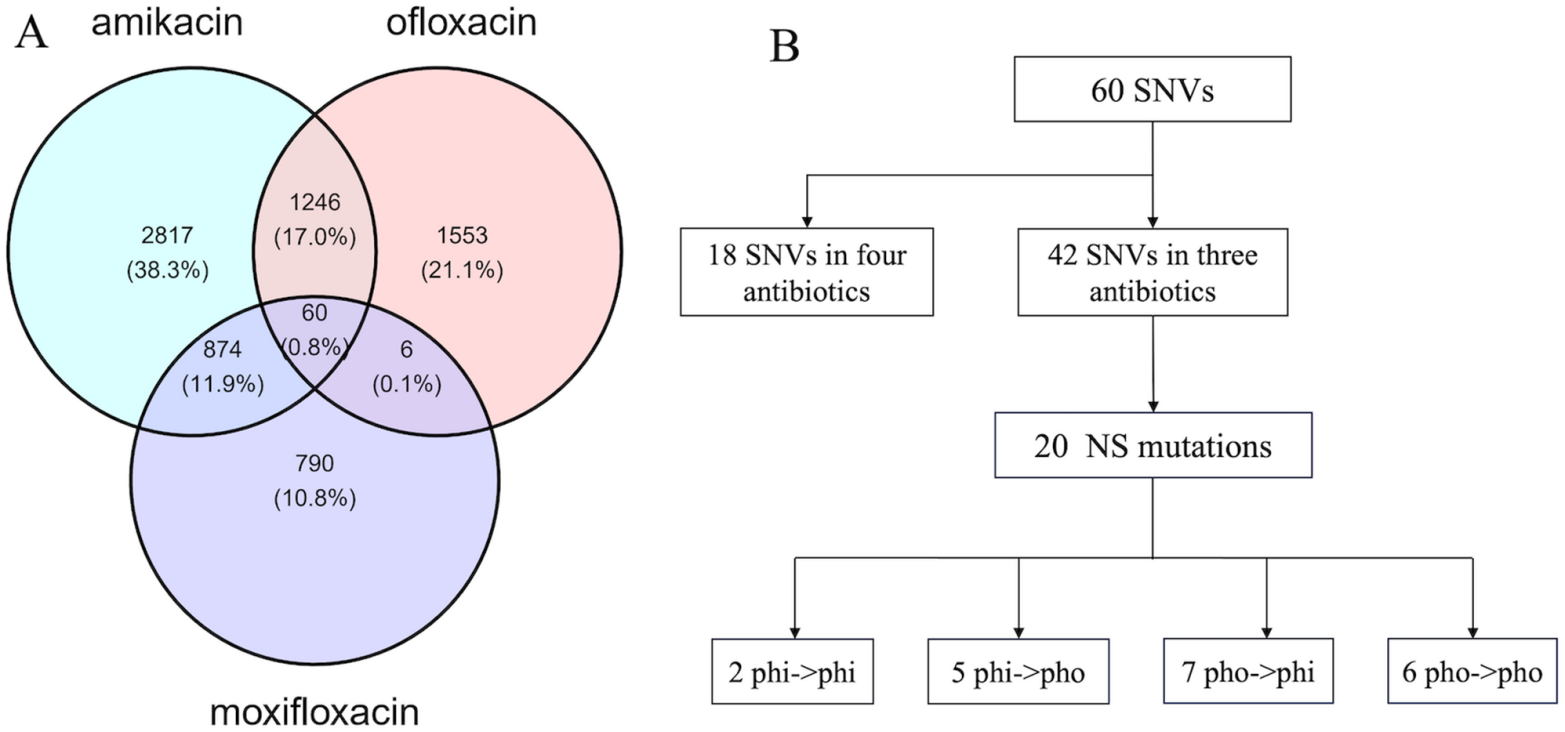
Common SNVs in XDR-TB with resistance to three antibiotics (moxifloxacin, ofloxacin, and amikacin). (**A**) Venn diagram of common SNVs in three antibiotics; (**B**) Hydrophilic and hydrophobic analysis of translated amino acid of common SNVs

The XDR-TB is defined as isolates that are resistant to first-line drugs (moxifloxacin and ofloxacin) and a least one second-line drug, i.e., the isolates resistant to three antibiotics (moxifloxacin, ofloxacin, capreomycin) or (moxifloxacin, ofloxacin, amikacin). In the case of XDR-TB resistant to moxifloxacin, ofloxacin, and capreomycin, we only found PE_PGRS4 and Rv0094c. In another case of XDR-TB resistant to moxifloxacin, ofloxacin, and amikacin, we identified more genes mutated, with a set of 60 significant SNVs (Fig. 5A and Table S1). Of the 60 SNVs, 51 were located within the coding region while the remaining nine were found in non-coding regions. This result indicated that the drug-resistance-associated SNVs were mostly enriched in the coding region of genes. In addition, 20 SNVs were NS mutations corresponding to 20 genes (ctpI, desA3, mce1R, moeB1, ndhA, PPE18, rpsA, secF, Rv0311, Rv0347, Rv0654, Rv0698, Rv0888, Rv0923c, Rv1431, Rv1672c, Rv1692, Rv1836c, Rv2028c, Rv3630). The details of these 20 genes are shown in Table 5. Out of these mutations, seven SNVs cause the coding AA to change from hydrophobic to hydrophilic, and other six SNVs do not change hydrophobic features (Fig. 5B). These results suggest that MTB proteins tend to mutate from hydrophilic to evade the hydrolysis by the host enzymes.

**Table 5:** Details of 20 genes in XDR-TB. The table did not include the PE_PGRS4 and Rv0094c, which are shown in Table 4. AA: amino acid; pho: hydrophobic; phi: hydrophilic

No.	Gene	Strand	RefSeq Position	Reference	Alternative	Condon Mutation	SNV Type	AA Mutation	Hydrophilic change
1	ndhA	-	472,148	T	G	CAT-> CAG	NS	His-> Gln	phi-> phi
2	Rv0888	+	988,005	A	G	AAC-> AGC	NS	Asn-> Ser	phi-> phi
3	desA3	-	3,606,019	C	T	ACG-> ATG	NS	Thr-> Met	phi-> pho
4	moeB1	-	3,583,542	A	C	ACG-> CCG	NS	Thr-> Pro	phi-> pho
5	PPE18	+	1,340,013	C	T	TCC-> TTC	NS	Ser-> Phe	phi-> pho
6	rpsA	+	1,833,909	A	C	GAC-> GCC	NS	Asp-> Ala	phi-> pho
7	Rv0698	+	798,915	A	C	CAG-> CCG	NS	Gln-> Pro	phi-> pho
8	mce1R	-	194,247	C	A	GCA-> GAA	NS	Ala-> Glu	pho-> phi
9	Rv0347	+	417,766	G	T	GGC-> TGC	NS	Gly-> Cys	pho-> phi
10	Rv0654	+	750,235	C	A	GCC-> GAC	NS	Ala-> Asp	pho-> phi
11	Rv0923c	-	1,030,418	G	A	GCC-> ACC	NS	Ala-> Thr	pho-> phi
12	Rv1672c	-	1,897,733	G	A	GGC-> AGC	NS	Gly-> Ser	pho-> phi
13	Rv2028c	-	2,274,673	G	A	GCG-> ACG	NS	Ala-> Thr	pho-> phi
14	Rv3630	+	4,069,757	T	C	TGG-> CGG	NS	Trp-> Arg	pho-> phi
15	ctpI	-	126,631	C	T	GCC-> GTC	NS	Ala-> Val	pho-> pho
16	Rv0311	+	380,097	C	T	GCC-> GTC	NS	Ala-> Val	pho-> pho
17	Rv1431	+	1,608,258	C	T	GCC-> GTC	NS	Ala-> Val	pho-> pho
18	Rv1692	+	1,917,506	C	T	CCG-> CTG	NS	Pro-> Leu	pho-> pho
19	Rv1836c	-	2,082,879	G	A	ATG-> ATA	NS	Met-> Ile	pho-> pho
20	secF	-	2,913,777	A	G	ATC-> GTC	NS	Ile-> Val	pho-> pho

### Three-dimensional protein structures of identified genes

Understanding three-dimensional (3D) structures of proteins is crucial for unraveling their functions and interactions in biological processes. We so far have identified 22 genes related to drug-resistance mechanisms in MTB, including 13 genes with only locus information (Rv0094c, Rv0311, Rv0347, Rv0654, Rv0698, Rv0888, Rv0923c, Rv1431, Rv1672c, Rv1692, Rv1836c, Rv2028c, Rv3630) and nine genes with more detailed information (ctpI, desA3, mce1R, moeB1, ndhA, PE_PGRS4, PPE18, rpsA, secF). We constructed the 3D structure of coding proteins for these nine genes by SWISS-MODEL and the parameters of these structures are shown in Table 6. Results showed the sequence identity scores with all templates were larger than 70%, indicating a high similarity between the template and our proteins. In particular, the structural templates of six proteins (PE_PGRS4, ctpI, moeB1, rpsA, ndhA, and PPE18) were identical to the homologous protein. The Global Model Quality Estimation (GMQE) value, is common index used for model quality evaluation in SWISS-MODEL. The GMQE values of all proteins are larger than 0.6 in 3D model. Specifically, the GMQE values of five coding proteins (PE_PGRS4, desA3, mce1R, moeB1, and ndhA) are larger than 0.9, suggesting the robust and reliable of AlphaFold method in the prediction of the three-dimensional structures in SWISS-MODEL.

**Table 6:** Summary of three-dimensional structures of proteins related to drug resistance mechanisms predicted by SWISS-MODEL. GMQE: Global Model Quality Estimation; AFDB: AlphaFold Protein Structure Database

Name	Template	Sequence identity	Oligo-state	GMQE	Found by	Method	Coverage
PE_PGRS4	L0T4W6.1.A	100	monomer	0.9	AFDB search	AlphaFold v2	1
ctpI	P9WPS5.1.A	100	monomer	0.74	AFDB search	AlphaFold v2	1
desA3	A0A1A0U1C5.1.A	85.48	monomer	0.94	AFDB search	AlphaFold v2	1
mce1R	A0A1X1U652.1.A	72.15	monomer	0.92	AFDB search	AlphaFold v2	0.98
moeB1	P9WMN6.1.A	100	monomer	0.93	AFDB search	AlphaFold v2	1
rpsA	P9WH43.1.A	100	monomer	0.71	AFDB search	AlphaFold v2	1
secF	P9WGN8.1.A	99.77	monomer	0.76	AFDB search	AlphaFold v2	1
ndhA	P95200.1.A	100	monomer	0.93	AFDB search	AlphaFold v2	1
PPE18	L7N675.1.A	100	monomer	0.69	AFDB search	AlphaFold v2	1

**Fig. 6:**
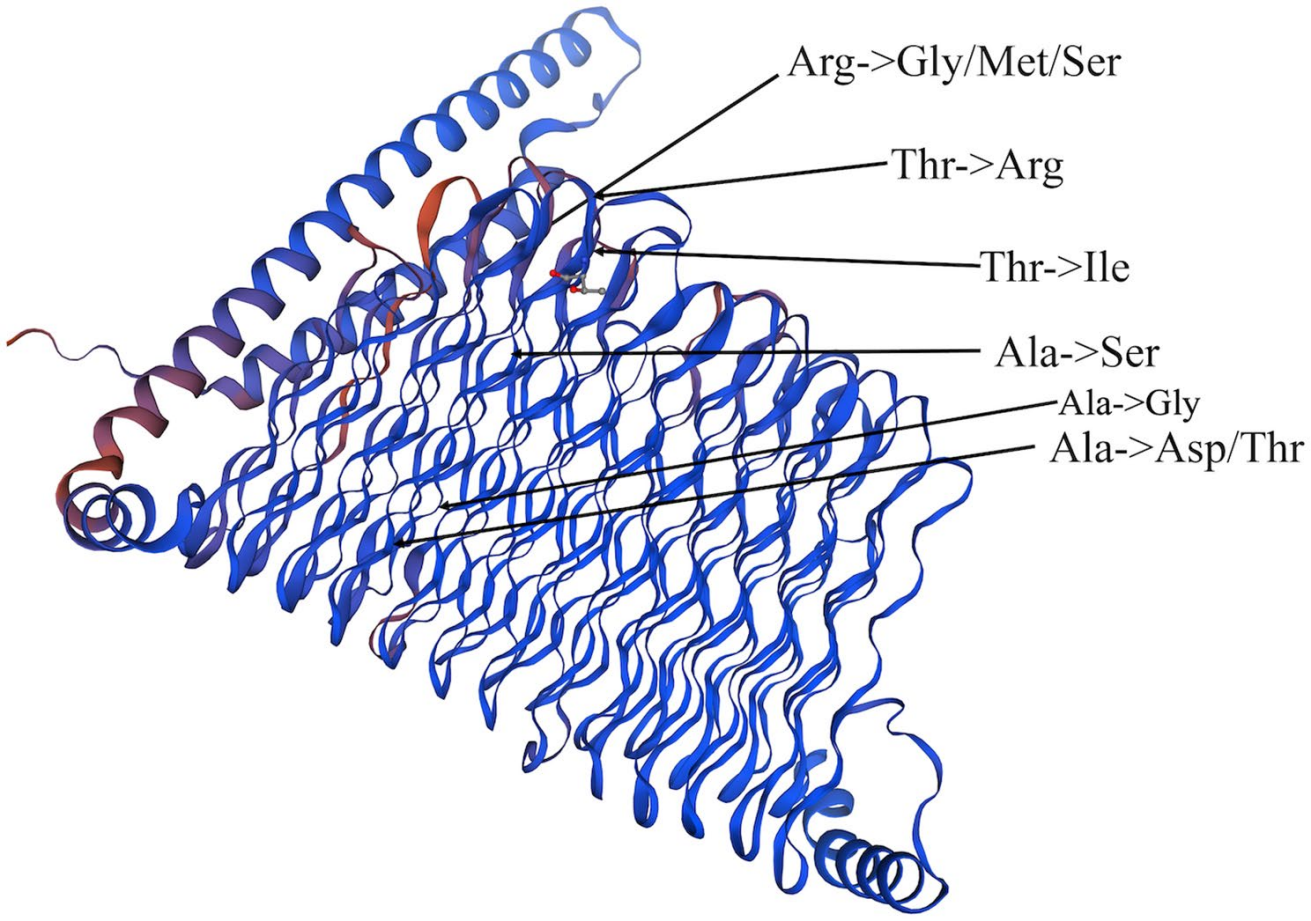
The positions of the amino acid mutation are caused by all non-synonymous SNVs in the 3D structure of the PE_PGRS4 protein

**Fig. 7:**
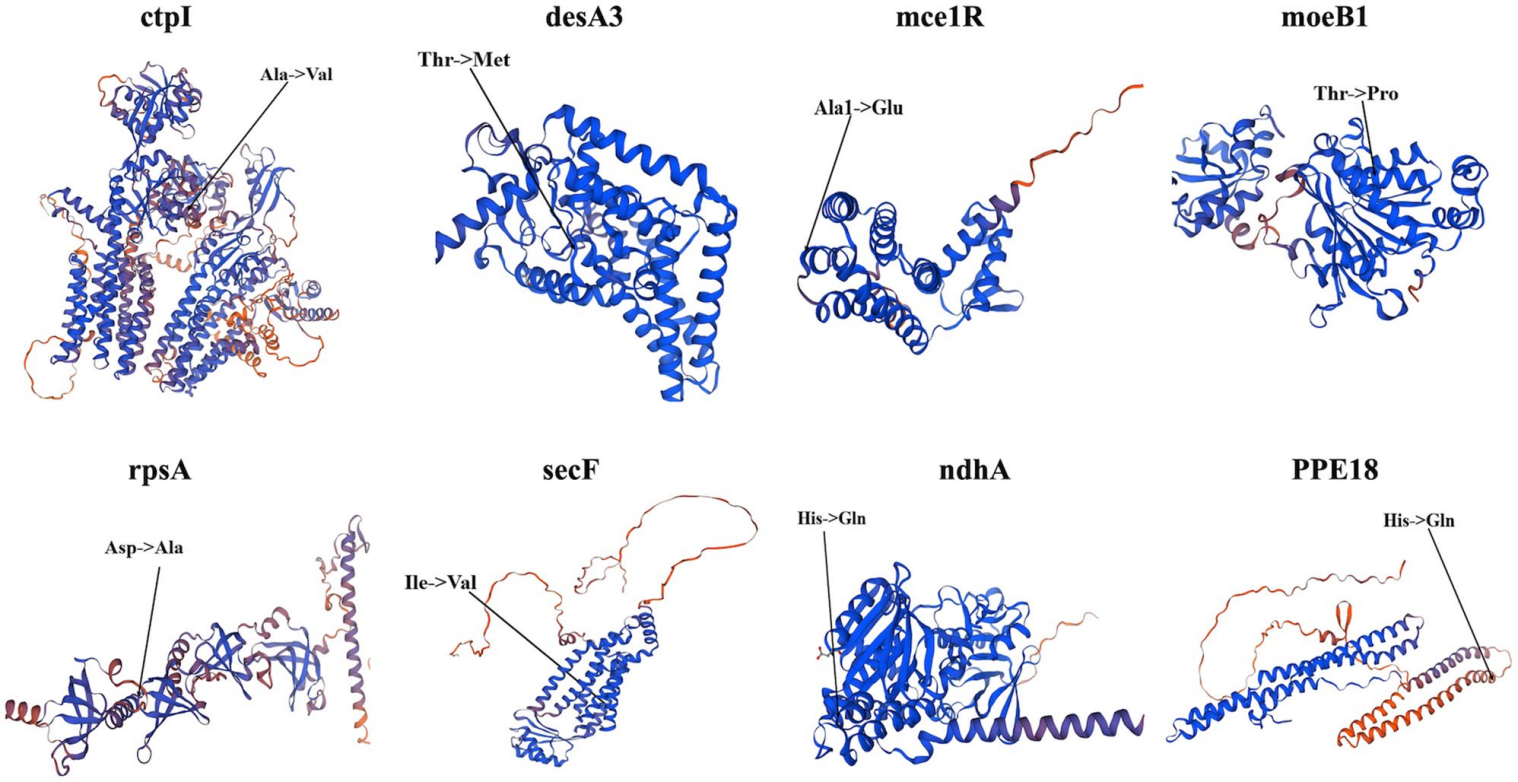
The positions of the amino acid mutations were caused by non-synonymous SNVs in 3D structure of the coding proteins of identified genes

Our analysis revealed four important SNV cluster regions within the PE_PGRS4 gene, highlighting its key role in various antibiotic-resistant strains. The 3D structure analysis showed that the SNV mutations of PE_PGRS4 were exclusively located within its β-folding region (Fig. 6). Results showed that Arg at position 174 exhibited three types of mutations (Arg-> Gly, Arg-> Met, and Arg-> Ser), while Ala at position 188 had two types of mutations (Ala-> Asp and Ala-> Thr). Additionally, Thr, Thr, Ala, and Ala at positions 177, 179, 182, and 196 experienced mutations to Arg, Ile, Ser, and Gly, respectively. The active site residue Ala is at position 196. Taken together, the amino acid positions from 174 to 188 in β-folding region of PE_PGRS4 gene were speculated to serve as the important catalytic sites in the protein function. In addition, the other eight genes found in isolates resistant to the other three antibiotics testing also displayed a solitary NS mutation, as illustrated in Fig. 7. Mutations in the five genes (ctpI, moeB1, rpsA, secF, PPE18) were situated within the α-helix structure, while mutations of rest genes (desA3, mce1R, and ndhA) located within the β-sheet structure.

### Identify critical active sites affected by SNVs

To uncover possible drug-resistance mechanisms, active sites of coding proteins of above genes were found by PrankWeb. The active sites affected by SNVs are shown in Table 7. Among these proteins, six (PE_PGRS4, desA3, mce1R, moeB1, secF, ndhA) of them were found at active sites nearby the position of amino acid mutations translated by SNVs. In particular, the position of mutated amino acids of three genes (PE_PGRS4, DesA3, secF) were closely near the active sites. The active site 172 of PE_PGRS4 was only two residues away from the mutation site 174. The mutation site 196 of desA is also the active site. The mutation site 79 of secF was located between active sites 77 and 78. These findings underscored the structural alterations induced by genetic mutations in key MTB genes, shedding light on potential implications for protein function and, by extension, drug resistance mechanisms.

**Table 7:** Active sites of coding proteins predicted by PrankWeb. Three proteins (ctpI, rpsA, PPE18) were not found active sites nearby mutated amino acids

Gene	UniProt ID of Protein	Position of mutation	Mutation	Position of active sites nearby
PE_PGRS4	L0T4W6	174	Arg-> Gly/Met/Ser	163, 165, 166, 168, 171, 172
PE_PGRS4	L0T4W6	179	Thr-> Ile	163, 165, 166, 168, 171, 172, 193, 195, 196, 198
PE_PGRS4	L0T4W6	182	Ala-> Ser	163, 165, 166, 168, 171, 172, 193, 195, 196, 198, 201, 202
PE_PGRS4	L0T4W6	188	Ala-> Asp/Thr	163, 165, 166, 168, 171, 172, 193, 195, 196, 198, 201, 202, 203
PE_PGRS4	L0T4W6	196	Ala-> Gly	193, 195, 196, 198, 201, 202, 203
ctpI	P9WPS5	1304	Ala-> Val	NA
desA3	P9WNZ3	339	Thr-> Met	320, 323,324, 327, 337, 339
mce1R	Q79G00	190	Ala-> Glu	170, 175
moeB1	P9WMN7	57	Thr-> Pro	48, 49, 51, 52, 53, 72, 73, 74, 75
rpsA	P9WH43	123	Asp-> Ala	NA
secF	P9WGN9	79	Ile-> Val	68, 72, 74, 75, 77, 78, 82, 87, 89, 91, 92, 93, 98
ndhA	P95200	164	His-> Gln	150, 169, 170, 176, 177, 179, 180, 181, 184
PPE18	L7N675	222	Ser-> Phe	NA

## Discussion

*Mycobacterium tuberculosis* is a bacterium that causes tuberculosis (TB), a potentially serious infectious disease that primarily affects the lungs. TB remains a significant global health concern, and efforts to control and eliminate the disease are ongoing. MTB is known for its slow growth rate compared to many other bacteria. However, MTB could be spread through the air when an infected person coughs or sneezes, releasing tiny droplets containing the bacteria [17]. Understanding the genome of MTB is essential for unraveling the mechanisms of its pathogenicity, drug resistance, and host interactions.

MTB have developed resistance to various drugs through a combination of genetic mutations and selective pressures [18]. The emergence of drug-resistant strains poses a significant challenge to TB control and treatment efforts. The drug resistance phenomenon could decrease the sensitivity of MTB strains and diminish the efficacy of available antibiotics [19]. This pathogen can carry antigenic mutation, allowing it to evade the immune response of host. Such mutations involve changes in surface antigens, making it more challenging for the immune system to recognize and eliminate the bacterium [20]. Understanding the mechanisms of drug resistance in MTB is crucial for developing new treatment strategies and improving the management of drug-resistant TB cases.

In this study, whole-genome sequencing (WGS) data of 716 clinical MTB isolates were analyzed for their drug-resistance mechanisms to two first-line drugs (moxifloxacin and ofloxacin) and two second-line drugs (amikacin and capreomycin). Moxifloxacin and ofloxacin are both fluoroquinolone antibiotics that target bacterial DNA synthesis and protein synthesis [21]. Amikacin inhibits translocation by binding peptide tRNA at the ribosomal A-site, thereby suppressing protein synthesis and rendering bacteria unable to survive [22]. Capreomycin, a ribosome-targeting peptide antibiotic, inhibits tRNA binding by interacting with the ribosome, thereby inhibiting protein synthesis [23]. We found a high proportion (94.2%) of testing isolates showed resistance to ofloxacin, indicating that this drug may not be suitable for the treatment of MTB. These MTB isolates were compared with H37Rv reference genome by MuMmer4 to identify SNVs. An average number of 3529 SNVs were observed per ofloxacin-resistant MTB isolate, with a mutation rate of around 0.08% under the selection pressure of ofloxacin.

As only a minority of SNVs influence MTB drug resistance mechanisms, Fisher’s exact test was employed to compare the mutation frequencies in the resistant and susceptible strains exposed to the four antibiotics (moxifloxacin, ofloxacin, amikacin, and capreomycin). At a significance threshold of p-value < 0.05, we found a total of 3536 and 3541 SNVs in moxifloxacin-resistant and ofloxacin-resistant MTB isolates respectively. The number of NS mutations is larger than that of the synonymous mutations in all four antibiotics.

To understand the resistance mechanisms in MTB, we examined 18 shared SNVs associated with these four antibiotics. Of the 18 SNVs, we found nine non-synonymous SNVs located in the coding region of PE_PGRS4 and Rv0094c. Previous study showed that the transcriptional expression profile of PE_PGRS4 was constitutively expressed and up-regulated under the circumstances of many antibiotics [24]. Besides, the mutations of PE_PGRS4 gene have been previously demonstrated in drug-resistant MTB [25]. Other SNVs in non-coding regions, such as A300922G, T3379432C, and T3381356C, may also have potential functional relevance. While these mutations may not directly impact protein coding, they could play a regulatory role in MTB. Regulatory variants have potential to exert a substantial influence on phenotype, emphasizing their significance in understanding the broader implications of these genetic mutations [26].

To further analyze the drug-resistant mechanism in XDR-TB, we identified a set of 60 shared SNVs related to three antibiotics (moxifloxacin, ofloxacin, and capreomycin). In addition to the 18 shared SNVs identified in MTB resistant to four antibiotics, we also identified 42 SNVs located in the coding region of eight genes (ctpI, desA3, mce1R, moeB1, ndhA, PPE18, rpsA, and secF). Previous studies indicated that these genes were highly related to drug-resistant mechanisms in XDR-TB, for example, rpsA, secF, and desA3. The rpsA protein plays a crucial role in translation initiation and mRNA binding during protein synthesis. Due to its essential role in protein synthesis, protein rpsA is considered a potential target for the development of antimicrobial drugs [27]. The secF protein is involved in the process of protein secretion across the bacterial inner membrane. The disruptions in protein secretion processes can potentially impact the physiology of the bacterium, thus the secF mutation is considered to be related to drug resistance in MTB [28]. The desA3 gene is associated with the biosynthesis of oleic acids, which is essential for the formation of the cell wall of actively replicating bacteria [29, 30]. The mutation of these genes may highly affect their function, which results in drug-resistant events in MTB.

We then constructed the 3D model of the coding protein of these nine genes. 3D structural analysis showed that SNV locations of three genes (PE_PGRS4, DesA3, and secF) were close to the active sites. These structural changes may have implications for protein structure and function, including protein stability, folding state, and interactions. For example, the mutations at position 174 are from arginine to glycine, resulting in the atom number also changed significantly. Specifically, arginine has six atoms on its side chain, while glycine has only one atom on its side chains. Previous studies showed that glycine-to-arginine mutations could cause conformational change and impaired transition metal transport in bacteria [31]. This result enhanced that the identified SNVs could be highly associated with drug-resistance mechanisms in MTB.

## Conclusion

In this study, we obtained 716 MTB isolates tested by four antibiotics and identified the mutated SNVs by MuMmer4. Fisher’s exact test was applied to whole-genome sequencing data from clinically relevant MTB isolates to identify SNVs related to drug resistance. Non-synonymous mutations within the PE_PGRS4 gene were found in strains resistant to four antibiotics, highlighting the potential of PE_PGRS4 as a biomarker of broad-spectrum resistance. Our exploration of XDR-TB isolates revealed distinctive roles of another eight genes (ctpI, desA3, mce1R, moeB1, ndhA, PPE18, rpsA, and secF) in resisting second-line drugs. This study illustrates the complexity of drug-resistant tuberculosis and emphasizes the significance of non-synonymous SNVs in MTB drug resistance. These findings not only advance our comprehension of MTB resistance mechanisms but also offer insights for targeted intervention development.

## Materials and methods

### WGS data retrieving and analysis

Whole-genome sequencing (WGS) data of 716 MTB isolates (Table S2) were obtained from the Bacterial and Viral Bioinformatics Resource Center (BV-BRC) database [32]. The MTB H37Rv reference genome was downloaded from the National Center for Biotechnology Information (NCBI) Genome database. The gene annotation file was also retrieved from the NCBI database.

### SNV calling by MuMmer4

SNVs are the most common type of genetic mutation among pathogens and are often used as biomarkers to study the association between genetic mutations and bacterial traits. These SNVs represent mutations arising from point mutations at precise gene locations, resulting in resistance against certain antibiotics in bacteria [33]. The WGS data of MTB isolates resistant to antibiotics were compared against the H37Rv reference genome using MUMmer4 to identify possible SNVs by the script ‘nucmer reference_file.fasta query_file.fasta out-file.delta show-snps -Clr out.delta’. MUMmer4 presents a notable performance in processing large genomes, enhanced speed, compatibility with scripting languages, and suitability for SNV calling [34].

### SNV screening by Fisher’s exact test

While most genomic mutations have minimal effects on phenotype, only a few SNVs were expected to strongly influence complex traits of bacteria [35]. In this study, a group of 716 strains were categorized as either resistant or sensitive to four antibiotics (moxifloxacin, ofloxacin, amikacin, and capreomycin). Subsequently, Fisher’s exact test was employed to compare the frequencies of resistant and non-resistant strains for each antibiotic and SNVs were screened at a threshold of statistical p-value < 0.05. Fisher’s exact test, which is recognized for its better accuracy with a small sample test, was chosen to assess the null hypothesis of SNVs in drug-resistant features [36]. For isolates resistant to moxifloxacin, ofloxacin, amikacin, and capreomycin, we identified 4996, 5026, 1730, and 2864 significant SNVs, respectively.

The formula for Fisher’s exact test is as follows:

$$\displaylines{P = {{\left( {\matrix{A \cr {A + C} \cr } } \right)\left( {\matrix{B \cr {B + D} \cr } } \right)} \over {\left( {\matrix{{A + B} \cr N \cr } } \right)}} = {{\left( {\matrix{B \cr {A + B} \cr } } \right)\left( {\matrix{D \cr {C + D} \cr } } \right)} \over {\left( {\matrix{{B + D} \cr N \cr } } \right)}} \cr = {{\left( {A + B} \right)!\left( {C + D} \right)!\left( {A + C} \right)!\left( {B + D} \right)!} \over {A!B!C!D!}} \cr}$$

**Table:** 

	Presence of a SNV	Absence of a SNV	Total
Resistant MTB strains	A	B	A + B
Non-resistant MTB strains	C	D	C + D
Total	A + C	B + D	A + B + C + D = N

A: The number of strains with a given SNV in resistant strains.

B: The number of strains without the given SNV in resistant strains.

C: The number of strains with the given SNV in non-resistant strains.

D: The number of strains without the given SNV in non-resistant strains.

### Common SNVs related to four antibiotics

SNV cluster regions, defined by the presence of multiple neighboring SNVs in the same or adjacent genomic regions, potentially influence MTB resistance [37]. We investigated the common resistance mechanism among the four antibiotic-resistant strains to identify shared SNVs among them. The genes associated with these SNVs were also identified based on their genomic location. Only PE_PGRS4 and Rv0094c were found in the SNVs related to four antibiotic-resistant isolates. Thus, the biological parameters of these two genes were further analyzed.

### Common genes in XDR-TB

The XDR-TB was defined as those cases resistant to all first-line drugs (mainly fluoroquinolones) and a least one second-line drug [5]. To further investigate the resistance mechanisms associated with XDR-TB, we analyzed the shared significant SNVs for two fluoroquinolones (moxifloxacin and ofloxacin) and one of second-line drugs (amikacin and capreomycin). Thus, the two cases of XDR-TB (two fluoroquinolones with ofloxacin, and two fluoroquinolones with capreomycin) were further analyzed. The involved transcribed amino acids and proteins of these genes were identified to distinguish synonymous and non-synonymous (NS) mutations.

### Construction of three-dimensional structures of identified proteins

Protein sequences, coupled with their three-dimensional (3D) structures, provide critical information for understanding protein functions, interactions, and biological processes [38]. In this study, three-dimensional structures of proteins with non-synonymous mutations were constructed by SWOSS-MODEL, the online server for automated protein homology modeling [39]. The SWISS-MODEL could incorporate features to predict the structure and stoichiometry parameters of complexes based on the amino acid sequences of interacting proteins. A novel modeling engine (AlphaFold) and a local model quality assessment method (QMEANDisCo) were applied to enhance the accuracy of protein modeling in SWISS-MODEL [40].

### Identify critical active sites affected by SNVs

Mutations in the active site can lead to dramatic changes in protein activity and affect the efficiency of binding drugs, thus identification of the active site is necessary. In this study, protein active sites were predicted by PrankWeb, which adopted deep learning model to characterize the binding site of protein and the ligand [41]. PrankWeb is a user-friendly web tool that allows users to enter a UniProt accession number as the input. The predicted active sites were then compared with the locus of amino acid changes caused by SNVs to identify critical active sites.

### Electronic supplementary material

Below is the link to the electronic supplementary material.

Supplementary Material 1

## Data Availability

All genome sequencing data for this analysis are available in the BV-BRC database (https://www.bv-brc.org/).

## Abbreviations

AA: Animo acid
TB: Tuberculosis
MTB: Mycobacterium tuberculosis
WGS: Whole-genome sequencing
NS mutation: Non-synonymous mutation
S mutation: Synonymous mutation
SNV: Single nucleotide variant
phi: Hydrophilic
pho: Hydrophobic
XDR-TB: Extensively drug-resistant tuberculosis
MDR-TB: Multidrug-resistant tuberculosis
NCBI: National Center for Biotechnology Information
BV-BRC: Bacterial and Viral Bioinformatics Resource Center
3D: Three-dimensional
GMQE: Global Model Quality Estimation
AFDB: AlphaFold Protein Structure Database
